# Surgical Management of Cervical Ossification of Posterior Longitudinal Ligament: The Treatment Algorithm and Outcome

**DOI:** 10.7759/cureus.36517

**Published:** 2023-03-22

**Authors:** Bing Wui Ng, Jin Aun Tan, Suffian Sabri, Azmi Baharuddin, Mohd Hisam Muhamad Ariffin

**Affiliations:** 1 Orthopaedics and Traumatology, Universiti Kebangsaan Malaysia (UKM) Medical Centre, Kuala Lumpur, MYS; 2 Orthopaedics and Traumatology, Hospital Pakar Kanak-Kanak Universiti Kebangsaan Malaysia (UKM), Kuala Lumpur, MYS

**Keywords:** anterior approach, neck disability, cervical myelopathy, posterior longitudinal ligament, ossification

## Abstract

Introduction

Managing patients who present with symptoms of cervical myelopathy secondary to cervical ossification of the posterior longitudinal ligament (OPLL) is challenging. Various factors such as the number of levels involved with OPLL, types of OPLL, canal occupying ratio, K-line characteristics, and C2-C7 lordosis angle were found to guide decision-making and surgical approaches in managing this condition. However, no clear treatment algorithm has been published. This study aims to investigate the outcome of the management of cervical OPLL using a treatment algorithm used in a tertiary university hospital.

Methods

This is a retrospective cross-sectional study. Patients with cervical myelopathy secondary to cervical OPLL who were treated surgically in our center from 2014 to 2020 were included in this study. Demographic data and preoperative parameters that determined the treatment given according to our treatment algorithm were analyzed.

Result

A total of 24 patients fit the inclusion and exclusion criteria of the study. The mean recovery rate for all groups is 61.8$\pm$21.9% and the mean postoperative neck disability index (NDI) is 17.83$\pm$16.67%. There was a statistically significant difference between preoperative and postoperative Japanese Orthopaedic Association (JOA) scores for both anterior and posterior surgery subgroups.

Conclusion

We believe that the treatment algorithm used in our center could benefit other surgeons as a guide in managing patients who suffer from cervical myelopathy secondary to cervical OPLL. Further study including newer techniques would increase the surgeon's arsenal in providing the best outcome in managing this condition.

## Introduction

Cervical ossification of the posterior longitudinal ligament (OPLL) is a degenerative condition characterized by abnormal calcification which occurs within the posterior longitudinal ligament at the cervical spine. The presence of large OPLL in this region could cause cervical spinal canal stenosis resulting in various degrees of neurological deficit [1]. Compression of the spinal cord could manifest as symptoms of cervical myelopathy, radiculopathy, or myeloradiculopathy. The presence of such symptoms is a strong indication of surgical decompression [2].

The prevalence of OPLL in the East Asian population ranges from 1.9% to 4.3% while a study in India found the prevalence rate of cervical OPLL in their country to be around 5.12% [3,4]. Genetic predispositions were found to be associated with the occurrence and severity of OPLL [5]. To date, OPLL is associated with diseases such as ankylosing spondylitis, diffuse idiopathic skeletal hyperostosis (DISH), and diabetes mellitus [6]. Due to its proximity to neurological structures, conservative treatment might be ineffective in advanced cases and surgical management of this condition poses significant challenges and risks. Possible complications related to surgery include spinal cord injury, durotomy, and complications related to instrumentation. Thus, deciding the most effective approaches is important in maximizing treatment outcomes.

Surgical management of patients with cervical OPLL with neurology could be largely divided into anterior, posterior, and combined approaches. Various studies have investigated factors that could influence the decision-making and approach towards surgical treatment of cervical OPLL such as preoperative cervical lordosis, number of levels involved with OPLL, K-line, space available for cord (SAC), canal occupying ratio, and anatomy of OPLL [3,7-9]. These radiographic parameters need to be considered carefully together with the clinical condition of the patients to come up with the best management strategy. Each approach, whether anterior or posterior, has advantages and limitations. Many studies focus on the comparison of techniques regardless of differences in OPLL where decision-making for these techniques was not clearly stated. To date, no clear treatment algorithm has been proposed. This study aims to investigate the outcome of patients undergoing surgical treatment for cervical OPLL based on the decision-making and surgical approaches outlined by a single-center treatment algorithm. 

## Materials and methods

This is a retrospective cross-sectional study. All patients who underwent surgical treatment to diagnose cervical myelopathy secondary to cervical OPLL in a tertiary university hospital in Malaysia from the period of January 2014 to December 2020 were included in this study. Exclusion criteria include patients with a history of trauma to the cervical spine, congenital spinal deformity, tumor, infection, metabolic bone disease, poorly controlled diabetes mellitus, neurological diseases, patients with clinical symptoms due to compression of the spinal cord in the thoracic or lumbar region, patients whose clinical examination does not correlate with the MRI findings, and patients with incomplete data. Medical records, surgical records, imaging studies, intraoperative video recordings from the microscope, and individual patients’ clinic notes were reviewed and analyzed. The parameters relevant to this study were divided into clinical and radiographic parameters. Clinical parameters which were assessed include demographic data, types of surgery, preoperative Japanese Orthopaedic Association (JOA) score [10], JOA score during follow-up, neck disability index (NDI) during follow-up [11], rate of recovery [12], and presence of complications. Radiographic parameters include preoperative C2-C7 lordosis angle, number of levels affected by OPLL, types of OPLL, presence of double-layer sign on CT images [13], and canal occupying ratio documented. These radiographic parameters were used to decide on the treatment approach, while the clinical parameters were compared to determine the functional outcome of the patients in response to treatment.

Treatment algorithm

Single/Dual Level OPLL

**Figure 1:**
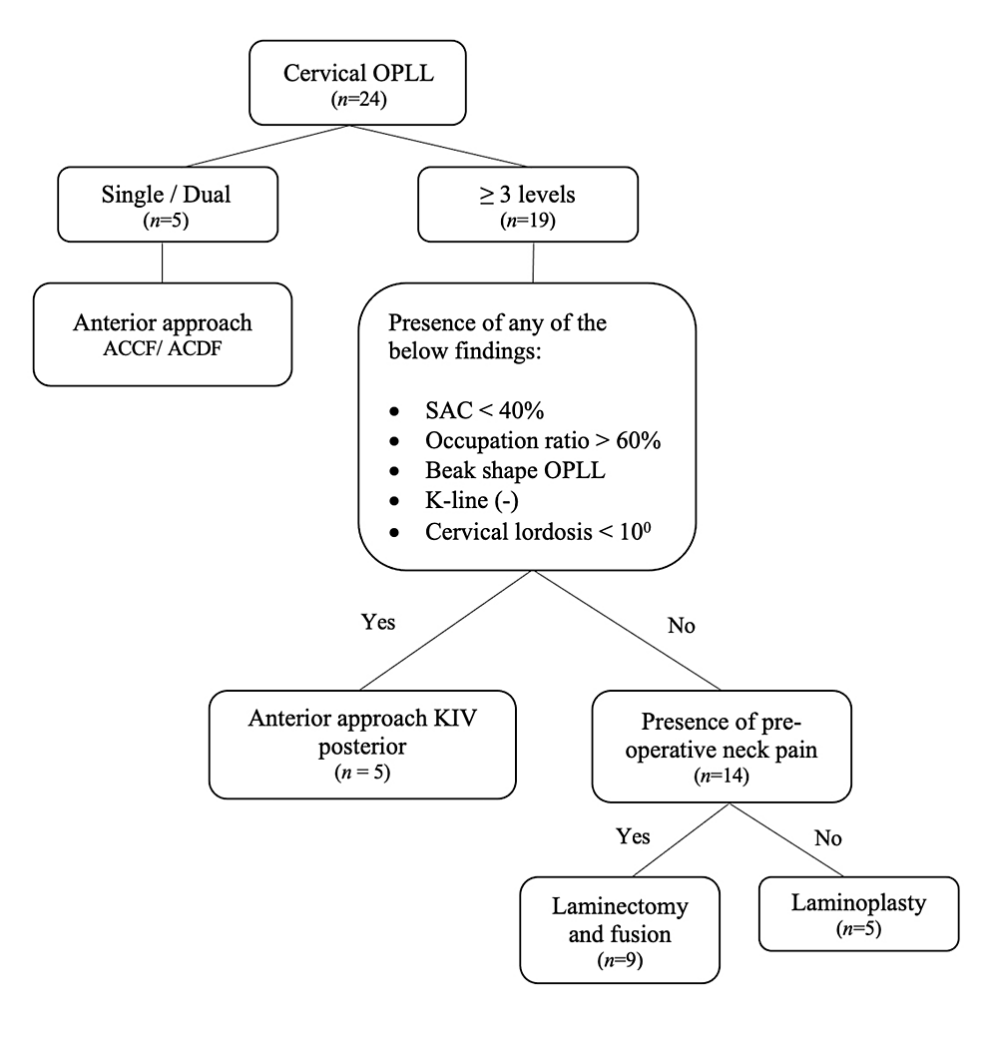
Treatment algorithm for decision-making of surgical treatment of cervical OPLL OPLL: Ossified posterior longitudinal ligament, ACCF:  Anterior cervical corpectomy and fusion, ACDF: Anterior cervical discectomy and fusion, SAC: Space available for cord, KIV: Keep in view

We outlined a treatment flow for the surgical treatment of cervical OPLL (Figure 1). Treatment options are first divided by the number of vertebrae involved with OPLL determined via CT images. For patients who suffered from single/dual level OPLL, the anterior approach was used to address the diseased region directly. Anterior cervical discectomy and fusion (ACDF) would be the ideal choice of treatment for local OPLL located at the corner of the endplate confined to the disc level. Patients with OPLL situated behind the vertebral body were managed with anterior cervical corpectomy and fusion (ACCF). We routinely used the digital microscope in anterior cervical surgery to enable better visualization, especially during the removal of OPLL adherent to the thecal sac. We did not perform the OPLL ‘floating' technique, and all OPLLs were removed as much as possible.

OPLL Involving Three or More Vertebrae

Other parameters are considered when deciding the surgical approach for patients who suffer from cervical OPLL which extends to three or more vertebrae. These factors include SAC of less than 40% on CT, canal occupying ratio of more than 60% on CT [9], beak-shaped OPLL, negative K-line [7], and C2-C7 cervical lordosis angle of less than 10 degrees [14]. The presence of any of these five factors would favor an anterior approach or a 360 degrees fusion surgery; otherwise, a posterior approach is utilized. If a posterior approach is in favor, the presence of preoperative neck pain will direct the decision toward posterior instrumentation and fusion. In contrast, the absence of neck pain would favor the decision to perform posterior cervical laminoplasty.

Surgical technique

**Figure 2:**
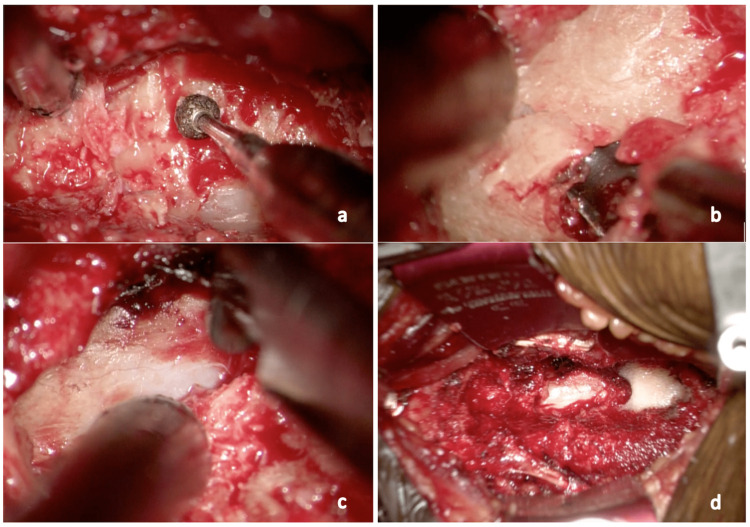
Intraoperative microscope pictures showing the thinning and removal of OPLL a: Thinning of OPLL via an anterior approach; b: Kerrison rongeur used to break off a mixed-type OPLL starting from the periphery. Note that despite a small fragment of OPLL having broken off from the main fragment, there was no expansion of the spinal cord; c: Removal of OPLL along the spinal cord; d: Complete removal of OPLL OPLL: Ossification of the posterior longitudinal ligament

**Figure 3:**
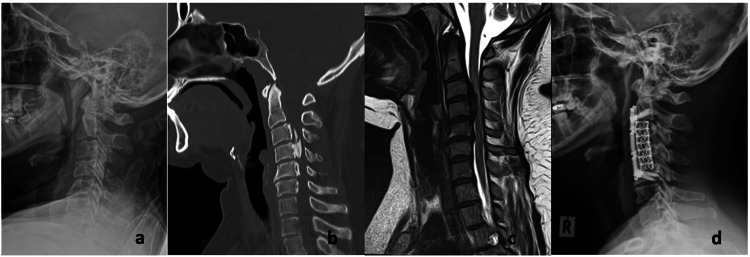
Representative case of a patient who has undergone anterior cervical corpectomy and fusion a: Plain radiograph showing neutral cervical lordosis; b: CT imaging with segmental OPLL involved from C3 to C5 and occupying ratio of more than 60%; c: MRI image showing spinal cord compression without hyperintensity of the spinal cord; d: Anterior cervical corpectomy of C3 and C4 OPLL: Ossification of the posterior longitudinal ligament

Anterior approach to the cervical spine was performed with the patient in a supine position via the Smith Robinson approach to the cervical spine. Anterior cervical surgeries were performed with the help of an exoscope, the Kinevo 900 (Carl Zeiss Meditec AG, Oberkochen, Germany) for its benefit of excellent illumination, magnification, visualization, and depth perception paramount in OPLL removal [15]. The OPLL which is visualized is thinned using a burr and lifted off with the help of microcurette and Kerrison Rongeur (Figure 2). Polyetheretherketone (PEEK) cages and an anterior cervical plate were used to stabilize the spinal segment. We routinely use a mesh cage filled with grafts obtained from corpectomy to reconstruct the anterior column if ACCF is performed (Figure 3).

**Figure 4:**
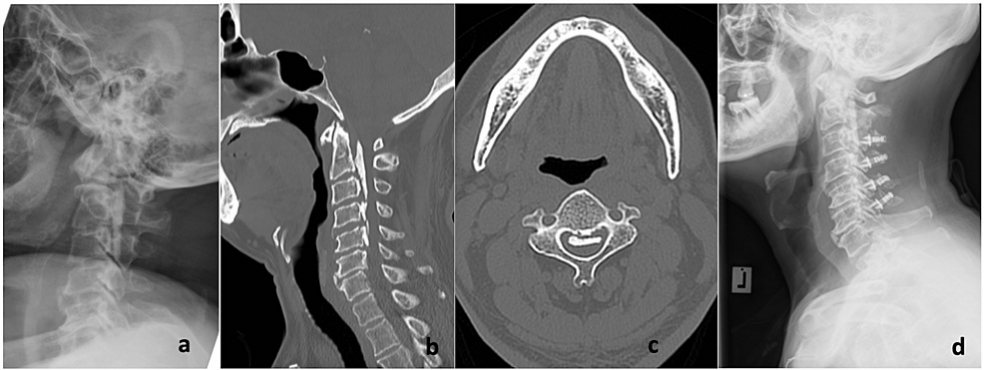
Patient with multiple-level cervical OPLL a: Plain radiograph showing the presence of cervical lordosis; b: Presence of multiple mixed-type cervical OPLL from C2 to C5; c: Occupying ratio is less than 60%; d: Laminoplasty was performed using a plate system OPLL: Ossification of the posterior longitudinal ligament

Posterior cervical surgery in this context consists of posterior decompression and fusion or posterior cervical laminoplasty. Both procedures were done with the patient in a prone position and the head immobilized with the help of a Mayfield head clamp. Posterior decompression and fusion were done with the insertion of lateral mass screws. A trough is created on both sides of the lamina, creating a dome-shaped cut at the cephalad and caudal ends of the area of interest. The posterior element which consists of the spinous process and lamina, is removed en bloc. If laminoplasty is to be performed, the trough is done on both sides of the lamina; however, only one side of the trough would be completed and elevated. We routinely use Centerpiece Plate Fixation System (Medtronic Sofamor Danek, Memphis, TN, USA) for our laminoplasty cases (Figure 4).

Data analysis

The demographic characteristics, presence of the mentioned confounding factors, and surgical approaches were tested as having a possible effect on the outcome. The effect of the treatment outcome was assessed by comparing patients' JOA scores before and after the surgery in each group. All clinical and radiographic parameters are described in terms of mean and standard deviation (SD) if the data is found to be normally distributed. Parametric data were analyzed using a one-way ANOVA test, while categorical data were compared using the chi-square test. Intergroup comparisons were made using the one-way ANOVA test. For non-parametric data, the Kruskal Wallis and Wilcoxon signed-rank tests were used. All statistical analysis was done using SPSS version 22 (IBM Corp., Armonk, NY, USA), and statistical significance was defined as p-value <0.05.

## Results

Demographic characteristics

A total of 34 patients who received surgical treatment between January 2014 to December 2020 for cervical OPLL were identified. From that, five patients were lost to follow-up, three patients had incomplete data, and two patients passed away due to reasons not related to cervical OPLL. That made a total of 24 patients who fulfilled the inclusion criteria for this study. Of the 24 patients, four (16.7%) were female, and 19 (83.3%) were male. The mean age of the study cohort is 63.33$\pm$14.46 years old and the mean follow-up duration is 42.33$\pm$28.43 months. Five patients had OPLL less than three levels and underwent anterior surgery. Nineteen patients were found to have OPLL of more than three levels. Out of these patients, five had one or more of the factors listed and had undergone anterior surgery. None of the patients received anterior and posterior stabilization. Twelve patients with no factors listed in the algorithm were further divided into the laminectomy and fusion group (n=7) and the laminoplasty group (n=5). Two patients with DISH and neutral cervical lordosis were included in the laminectomy and fusion group.

Clinical and radiographic characteristics

A statistical normality test was done and all data was found to be normally distributed. The type of OPLL was categorized into local (12.5%), segmental (33.3%), continuous (4.2%), and mixed (50.0%) based on the classification discussed in a previous study [16]. Eighteen patients (75%) had K-line positive while only six patients (25%) had K-line negative. Double-layer sign was present in 10 patients (41.7%). The mean occupying ratio noted on CT images was 50.4$\pm$10.64%. Only three patients (14%) had an occupying ratio of more than 60%. The mean C2-C7 angle was 8.87$\pm$15.63 degrees. The mean preoperative JOA score was 9.2$\pm$3.84. Each operative group's preoperative clinical and radiological parameters were summarized (Table 1). There was no statistically significant difference in age, gender, follow-up period, preoperative C2-C7 lordosis angle, and preoperative JOA score among the operative groups.

**Table 1:** Demographic, preoperative clinical and radiological characteristics of patients based on groups outlined by the treatment algorithm ^a ^One-way ANOVA test, ^b ^chi-square, ^c ^Kruskal-Wallis test SD: Standard deviation, JOA: Japanese Orthopaedic Association, KIV: Keep in view, M: Male, F: Female

Groups	Anterior	Anterior KIV Posterior	Laminectomy and Fusion	Laminoplasty	p-value
Number (n)	5	5	9	5	-
Age	60.8$\pm$14.6	55.6$\pm$16.5	62$\pm$13.8	76$\pm$6.6	0.134^a^
Gender	5M	2M 3F	7M 2F	5M	0.037^b^
Follow-up, (months)	32.8$\pm$34.0	21.8$\pm$16.6	55.4$\pm$27.8	48.8$\pm$24.2	0.144^a^
Pre-operative C2-C7 lordosis angle (degrees, SD)	8.3$\pm$15.1	4.7$\pm$18.1	12.5$\pm$7.5	16.5$\pm$19.9	0.133^a^
Pre-op JOA score (mean, SD)	7.7$\pm$5.3	8.1$\pm$4.6	9.7$\pm$3.4	10.8$\pm$1.6	2.193^c^

Clinical outcome

The mean duration of operating time was 115.87$\pm$ 51.43 minutes. The median postoperative JOA score was 13.8$\pm$2.06. The mean NDI percentage was 17.83$\pm$16.67% and the mean recovery rate 61.8$\pm$21.9% (Table 2). There was no statistically significant difference between operating time, postoperative JOA score, recovery rate, and NDI percentage among the operative groups. We found no statistically significant difference between clinical outcomes and the approaches used. The statistically significant differences can be noted in each operative group's preoperative and postoperative JOA scores shown in Table 3.

**Table 2:** Post-operative outcome comparisons between groups ^a ^One way ANOVA test, ^b ^Kruskal-Wallis test SD: Standard deviation, JOA: Japanese Orthopaedic Association, KIV: Keep in view, NDI: Neck disability index

Groups	Anterior	Anterior KIV Posterior	Laminectomy and Fusion	Laminoplasty	p-value
Duration of operation (min)	118 $\pm$35.82	150$\pm$47.61	116$\pm$64.84	86$\pm$29.45	0.343^a^
Postoperative JOA (mean, SD)	14.1$\pm$2.3	14.1$\pm$2.5	13.8$\pm$2.3	13.4$\pm$0.9	0.349^b^
Recovery rate (percentage)	69.6$\pm$12.5%	70.3$\pm$23.5%	64.4$\pm$24.0%	40.7$\pm$12.8%	0.097^a^
NDI (percentage)	22.0$\pm$26.2	11.6$\pm$17.1	18.0$\pm$15.8	19.2$\pm$6.4	0.798^a^

**Table 3:** Comparison of JOA-score before and after surgery in each operative group ^a ^Wilcoxon signed rank test, * *p *<0.05 SD: Standard deviation, JOA: Japanese Orthopaedic Association, KIV: Keep in view

Outcome Measures	Preoperative JOA Score (mean SD)	Postoperative JOA Score (mean SD)	p-value
Anterior approach	7.7$\pm$5.34	14.1$\pm$2.35	0.043*
Anterior approach KIV posterior	8.1$\pm$4.63	14.1$\pm$2.53	0.043*
Laminectomy and fusion	9.7$\pm$3.47	13.8$\pm$2.37	0.009*
Laminoplasty	10.8$\pm$1.64	13.4$\pm$0.96	0.042*

Complications

No cases of dura leak, durotomy, or revision surgery were noted in this cohort. One patient who underwent ACCF had a proximal screw pullout of 50%; however, he did not suffer from any neurological deterioration or neck pain. No revision surgery was offered to the patient.

## Discussion

Various systematic reviews and meta-analyses have discussed the effect of anterior versus posterior approaches in treating cervical myelopathy secondary to OPLL. Most of the literature discussed each approach, comparing treatment outcomes between groups without in-depth discussion on decision-making pathways and patient selection methods. This study shows the thought process and algorithm to guide surgical treatment for various forms of cervical OPLL (as shown above in Figure 1).

Factors considered for decision-making incorporated in this treatment algorithm have been widely discussed in the literature. Anterior surgery was found to address the OPLL directly and enable its removal in its entirety; however, it was also associated with more complications [17]. Anterior cervical corpectomy and fusion produced better surgical outcomes for patients with an occupying ratio of 50% to 60% [9,14]. Patients who suffered from cervical OPLL of more than three levels and had undergone anterior surgery made up the highest neurological recovery rate in our cohort which is up to 70.3$\pm$23.5%. This explains that patients who suffered from cervical myelopathy resulting from more than three levels of OPLL but with one or more confounding factors present have the most significant neurological and functional recovery after anterior surgery.

Meta-analysis shows that anterior surgeries have a high complication rate of neurological deterioration, CSF leakage, and pseudoarthrosis compared to posterior surgeries [18,19]. The presence of the double-layer sign is almost always followed by CSF leakage. Yang et al. reported 15% of dural tears in patients with double-layer signs visualized on CT imaging in their study [20]. No CSF leakage was found in our cohort despite 43.5% of the patients showing double-layer signs on CT images. All OPLL in patients undergoing anterior surgery was thinned and removed as much as possible. We do not routinely perform the OPLL floating technique due to the possibility of incomplete ‘floating’ or impingement of the OPLL to the surrounding structures. Toshitaka et al. noticed the potential risks for incomplete decompression due to the incomplete floating technique in their cohort and suggested intra-operative CT to better assess the extent of decompression [21]. This technology could also provide informative feedback to improve performance. We believe that employing intraoperative navigation coupled with the navigated burr for difficult cases could better assess the depth of the OPLL and facilitate its complete removal. However, our experience using both modalities is still in its infancy. Furthermore, proximal screw pull-out was noted in a patient who underwent ACCF for OPLL involving more than three levels of vertebrae and an occupying ratio of more than 60%. There is a need for further study in this group of patients to determine the factors that can guide the decision to either only an anterior surgery or an anterior-posterior fusion surgery. In retrospect, adding on posterior instrumentation could further enhance stability and potentially prevent complications for this patient.

Posterior surgery such as laminectomy and laminoplasty has been known to be a workhorse in treating multiple-level cervical OPLL. Literature reported that laminectomy and fusion are better in maintaining cervical lordosis and recommended that laminoplasty be performed on patients with K-line (+), while laminectomy and fusion are for patients with K-line (-) [22,23]. However, some authors also reported that anterior and posterior approaches could significantly improve clinical outcomes. No statistical difference was found between the approaches to indicate the superiority of one another [17,23]. This has led us to believe that each technique and approach would be beneficial depending on careful patient selection. Studies have shown that laminoplasty and laminectomy with fusion could improve clinical outcomes. Stephens et al. reported that carefully selected patients without predominant pre-operative axial neck pain with normal to lordotic cervical spine could achieve good clinical outcomes post-laminoplasty [24]. In our study, laminoplasty and laminectomy were performed in patients with K-line positive; however, the selection criteria were based on pre-operative neck pain. Both groups achieved statistically significant clinical and functional improvement with this selection criteria. Despite having a higher mean NDI percentage when comparing posterior groups to anterior groups, there was no significant difference in NDI between both posterior subgroups.

Two patients from our series have to undergo posterior surgery despite having a C2-C7 lordosis angle of less than 10 degrees due to multiple levels of ossified anterior longitudinal ligament (OALL), making the anterior approach difficult. Murayama et al. reported a case of OPLL with OALL in a DISH patient successfully treated with posterior laminectomy and fusion with OALL excision and interbody fusion from the anterior approach [25]. Both of our patients have undergone posterior laminectomy and fusion only. They have shown significant neurological and functional improvement with minimal neck pain (NDI < 10%), most probably resulting from the fused cervical spine due to the anterior syndesmophytes. We believe by doing a posterior surgery only, we could minimize the potential complications which could be caused by the excision of OALL and anterior surgery. A longer follow-up will be needed to further explore the clinical outcome difference between posterior-only or anteroposterior surgery in patients who suffered from OPLL and DISH.

In our study, all patients from four groups experienced clinical improvement with a mean recovery rate of 61.9%. All patients regained upper limb function to a score of at least three in the JOA score. Further data analysis has shown that only two out of all the patients cannot walk without a cane or support. Out of five patients who suffered from bladder dysfunction, only one did not recover to normal bladder function during follow-up. Most patients still experience numbness of the upper limb, followed by numbness of the lower limb and trunk.

Newer techniques such as antedisplacement and fusion or vertebral body sliding osteotomy of the cervical spine for treating OPLL have been described [26,27]. The studies found these techniques to be more effective in cases with an occupying ratio of more than 60% and K-line negative. Atul et al. described the "only spinal fixation" technique in treating cervical myelopathy related to OPLL [28]. We have limited experience regarding these procedures thus, these techniques were not included in our treatment algorithm.

The main limitation of this study is the small sample size and limited follow-up period. However, the decision-making algorithm used in our study is largely based on published evidence on each approach in treating cervical myelopathy secondary to cervical OPLL.

## Conclusions

The authors believe that the treatment algorithm described in this study could guide choosing the appropriate surgical approach and patient to ensure the best neurological outcome. Each technique has been compared and shown no superiority over the others; however, the appropriate approach must be chosen based on patient and disease characteristics. We found that patients who suffered from more than three levels of OPLL could still achieve good neurological recovery with anterior approach surgery. This treatment algorithm could be further expanded in the future to accommodate newer surgical techniques.
